# Plant-Based Foam Stabilizers Based on Rice Protein Isolate-Sodium Alginate Composite Particles: Structure, Interfacial Properties, and Foam Performance

**DOI:** 10.3390/foods15213880

**Published:** 2026-10-29

**Authors:** Fuchao Zhan, Shiyu Xie, Songze Li, Yiming Wang, Yating Yu, Guangzhe Gao, Yimeng Liu, Xiaorong Zhang

**Affiliations:** 1 Sanya Institute of Nanjing Agricultural University, College of Food Science and Technology, Nanjing Agricultural University, Nanjing 210095, China; 2 Guizhou Key Laboratory of New Quality Processing and Storage of Ecological Specialty Food, Guiyang 550025, China; 3 Collaborative Technology Service Center for the High-Value Processing of Green Agricultural Products (Prepared Foods) in the Yangtze River Delta Region, Suzhou University, Suzhou 234000, China

**Keywords:** rice protein isolate, sodium alginate, interactions, particle properties, foaming properties

## Abstract

To address the low solubility and inferior interfacial activity of rice protein isolate (RPI), RPI/sodium alginate (SA) composite colloidal particles were fabricated via a facile heat-induced co-assembly. Incorporating SA stabilized the flexible, unfolded conformation of RPI while forming an outer hydrophilic shell, as evidenced by a reduction in surface hydrophobicity and an increased negative zeta potential. Consequently, both foamability and foam stability followed a bell-shaped trend with increasing SA proportion, peaking at RPI/SA mass ratios of 1:0.3 and 1:0.4. At these optimal ratios, the composites exhibited a minimum polydispersity index (0.20), a highly negative zeta potential (−43 mV), moderate apparent particle size (~1500 nm), and the highest interfacial dilatational modulus (~18–24 mN/m). Mechanistically, this balanced architecture facilitated rapid interfacial adsorption and subsequent molecular rearrangement at the air-water interface. Once adsorbed, the particles synergistically enhanced foam stability through robust electrostatic repulsion and steric hindrance—imparted by their surface charges and the hydrophilic SA shell—effectively suppressing bubble coalescence. Conversely, an excess of SA rendered the particles overly hydrophilic, hindering interfacial adsorption and impairing foaming performance. This work clarifies the composition–structure–foaming property relationships of RPI/SA composites and offers fundamental support for utilizing rice protein as a plant-derived foaming agent.

## 1. Introduction

Food foams are typical air-water colloidal dispersion systems widely utilized in mousses, ice creams and baked goods to impart a light texture, delicate mouthfeel and favorable flavor release. Thermodynamically, foams are metastable systems driven toward destabilization via gravitational drainage, bubble coalescence, and Ostwald ripening [1,2,3]. Consequently, effective stabilization requires surface-active components that lower interfacial tension and form a robust, viscoelastic interfacial film. While animal-derived proteins traditionally dominate as foaming agents, the escalating consumer demand for sustainable, clean-label, and allergen-free ingredients has catalyzed extensive research into high-performance plant-based alternatives [4,5].

Proteins serve as the core surface-active components in food foams, and their foaming functionality depends on sequential interfacial events: bulk diffusion, surface adsorption, conformational rearrangement, and interfacial film formation [6,7]. An ideal foaming protein needs high molecular flexibility for rapid interfacial anchoring and the ability to construct a cohesive, viscoelastic interfacial network through non-covalent interactions (such as hydrophobic interactions and hydrogen bonding) as well as disulfide cross-linking [8]. In contrast to animal proteins, however, most native plant proteins adopt compact globular conformations with limited aqueous solubility, which severely impedes interfacial unfolding and the formation of a continuous adsorbed layer, ultimately compromising foamability and foam stability [9,10].

Rice, as one of the most widely cultivated staple crops, generates abundant protein-rich by-products, and improving its high-value utilization aligns with the sustainable development of agri-food resources [11,12]. Among these, rice protein isolate (RPI) is a hypoallergenic plant protein with a well-balanced amino acid profile, holding great promise for infant formula, sports nutrition, and medical foods [13]. RPI mainly comprises albumin (5–10%), globulin (7–17%), prolamin (3–6%), and glutelin, which accounts for over 75–81% of the total protein [14,15,16]. Glutelin primarily exists as high-molecular-weight aggregates cross-linked by intermolecular disulfide bonds between acidic and basic subunits [17]. Due to this densely cross-linked, hydrophobic architecture, RPI shows extremely low solubility (2–4% at pH 4–6) and readily forms dense aggregates [14,18]. These structural constraints severely hinder protein migration toward the air–water boundary and subsequent interfacial relaxation, resulting in foaming performance inferior to animal and most legume proteins [19].

Various modification strategies—including physical processing (ultrasound, high pressure, and thermal treatment), enzymatic hydrolysis, and chemical derivatization—have been applied to improve RPI functionality [20,21,22]. Although these methods can enhance foamability to some extent, they often suffer notable limitations. For example, excessive proteolysis yields short peptides unable to form viscoelastic interfacial films, whereas physical or chemical treatments can induce uncontrolled macro-aggregation and a subsequent loss of interfacial activity [23,24]. Therefore, developing strategies that simultaneously promote rapid interfacial adsorption and reinforce the mechanical integrity of the resulting interfacial film remains a critical challenge.

Fabricating protein-polysaccharide colloidal complexes offers a promising route to overcome the functional barriers of plant proteins [25]. Sodium alginate (SA), a natural anionic linear polysaccharide from brown algae, consists of β-D-mannuronic acid (M) and α-L-guluronic acid (G) residues linked by 1→4 glycosidic bonds. Its abundant carboxylate groups confer excellent water solubility, film-forming capacity, and biocompatibility. Compared to other anionic polysaccharides such as pectin or κ-carrageenan, SA possesses a unique block copolymer sequence that imparts exceptional chain flexibility and localized electrostatic binding capability, making it uniquely suited for modulating the conformational flexibility of proteins and constructing highly viscoelastic networks [26,27,28]. Depending on environmental conditions, RPI and SA can interact through a synergy of localized electrostatic attraction, hydrogen bonding, and hydrophobic interactions, co-assembling into soluble complexes or colloidal particles [29,30]. SA incorporation provides dual benefits: steric hindrance and electrostatic repulsion suppress protein aggregation and improve colloidal dispersibility, while upon interfacial adsorption, the outward-facing hydrophilic SA chains elevate interfacial film viscoelasticity and local continuous-phase viscosity, effectively retarding bubble coalescence and Ostwald ripening [31,32,33,34,35].

Despite considerable progress on protein-polysaccharide systems for bulk gelation or oil–water interfacial stabilization (e.g., Pickering emulsions), a specific research gap remains regarding their dynamic behavior at the air-water interface [36]. Foam interfacial dynamics differ fundamentally from those of emulsions due to the significantly higher interfacial free energy and faster destabilization rates of gas bubbles [37]. Furthermore, most documented plant protein–polysaccharide complexes have centered on legume proteins (e.g., soy and pea) constructed via conventional electrostatic complexation [38,39]. In contrast, the high-insolubility matrix of RPI and the synergistic combination of ultrasonic disruption with staged heat-induced co-assembly remain largely unexplored. It remains unclear how the fine-tuned colloidal architecture of RPI/SA composite particles governs their adsorption kinetics, structural relaxation, and rheological network formation at the air–water boundary, nor has the compositional turning point for maximizing foam properties been identified.

In this study, RPI/SA composite colloidal particles were fabricated at varying mass ratios via ultrasonic disruption coupled with staged heat-induced co-assembly (at pH 5.9). Colloidal characteristics—including particle size, zeta potential, and surface hydrophobicity—were comprehensively evaluated to assess particle stability and interfacial affinity. Conformational transitions were probed using UV–vis and intrinsic fluorescence spectroscopy. Crucially, dynamic air–water interfacial properties (adsorption kinetics and dilatational rheology) were systematically examined to decipher the interfacial film-forming behavior of the composites. Finally, foamability and foam stability were quantified to establish the structure–composition–property relationships. This work elucidates the interfacial stabilization mechanisms of RPI/SA colloidal particles and provides a fundamental theoretical framework for designing high-performance, clean-label plant-based foam stabilizers.

## 2. Materials and Methods

### 2.1. Materials and Reagents

Broken rice flour was purchased from a local supermarket (Nanjing, China). Analytical-grade sodium alginate (SA), sodium dihydrogen phosphate dihydrate, disodium hydrogen phosphate dodecahydrate, hydrochloric acid (HCl), and sodium hydroxide (NaOH) were purchased from Sinopharm Chemical Reagent Co., Ltd. (Shanghai, China). 8-Anilino-1-naphthalenesulfonic acid (ANS) was obtained from Aladdin Biochemical Technology Co., Ltd. (Shanghai, China). Ultrapure water (>18.2 MΩ⋅cm) was used in all experiments.

### 2.2. Extraction of Rice Protein Isolate (RPI)

Rice protein isolate (RPI) was prepared by alkaline extraction followed by isoelectric precipitation according to the method of Dai et al. [13] with minor modifications. Briefly, broken rice flour was dispersed in ultrapure water at a solid-to-liquid ratio of 1:8 (*w*/*v*), and the suspension was adjusted to pH 12.0 using 2 mol/L NaOH. The mixture was stirred at room temperature for 2 h to solubilize the proteins and then centrifuged at 7000× *g* for 25 min at 4 °C. To maximize protein recovery, the insoluble residue was re-extracted twice under identical conditions, and the supernatants from the three consecutive cycles were pooled. The combined supernatant was adjusted to pH 4.8 with 2 mol/L HCl to precipitate the proteins at their isoelectric point. The resulting precipitate was collected by centrifugation (7000× *g*, 25 min, 4 °C), redispersed in ultrapure water, and neutralized to pH 7.0 with 2 mol/L NaOH. The neutralized suspension was transferred into dialysis bags (molecular weight cutoff, MWCO 3500 Da) and dialyzed against ultrapure water at 4 °C for 48 h, with the external water replaced every 8 h to remove residual salts. The dialyzed suspension was freeze-dried, and the resulting RPI powder was sealed in airtight bags and stored at −20 °C until further use. The protein content of the extracted RPI powder was determined to be 92.36 ± 1.02% (N × 5.95) using the Kjeldahl method, and the extraction yield was approximately 10% based on the initial protein content of the broken rice flour.

### 2.3. Preparation of RPI/SA Composite Particles

An RPI stock solution (4%, *w*/*v*) was prepared by dispersing RPI powder in ultrapure water, adjusting the pH to 12.0 with 2 mol/L NaOH, stirring magnetically at 25 °C for 2 h, followed by hydration at 4 °C for 12 h. The SA stock solution (2%, *w*/*v*) was prepared by dissolving SA in ultrapure water under magnetic stirring, followed by hydration at 4 °C for 12 h to ensure complete dissolution.

Appropriate volumes of the two stock solutions were mixed according to the formulations listed in Table 1 and diluted with ultrapure water to a final volume of 10 mL. The final RPI concentration was fixed at 1.0% (*w*/*v*), and final SA concentrations ranged from 0% to 0.5% (*w*/*v*). The mixture’s pH was temporarily adjusted to 12.0 with 2 mol/L NaOH and stirred for 1 h to ensure homogeneous blending. The pH was then slowly titrated to 5.9 with 0.1 mol/L HCl under continuous stirring. This specific pH of 5.9 (slightly above the isoelectric point of RPI, pH ~4.8) was selected to prevent macroscopic precipitation of RPI while enabling localized electrostatic interactions between positively charged patches on RPI and fully ionized carboxyl groups on SA. The dispersions were then subjected to a two-stage thermal treatment (65 °C for 10 min, followed by 80 °C for 20 min) in sealed tubes within a circulating water bath to induce co-assembly. The system pH remained stable at 5.9 ± 0.1 throughout the heat treatment. The samples were immediately cooled in an ice-water bath for 5 min to terminate thermal assembly. Finally, to disrupt oversized aggregates and ensure a uniform particle size distribution, the dispersions were ultrasonicated using a probe ultrasonic processor (FB705, Fisher Scientific, Waltham, MA, USA; 6 mm tip) at 30% amplitude (~60 W) with a pulse mode (1 s on/1 s off) for 20 min in an ice bath.

### 2.4. Turbidity Measurement

The turbidity of the RPI-SA composite particle dispersion was determined according to our previously described method [40]. Prior to analysis, the samples were diluted 100-fold with ultrapure water (pre-adjusted to the corresponding pH) to bring the readings within the linear range of the instrument. The transmittance (T) of the diluted samples was measured at 600 nm using a microplate spectrophotometer (UV-1100, MAPDA, Shanghai, China) with ultrapure water as the blank. The turbidity was calculated as follow:

Turbidity = 100 − T%

where T represents the transmittance at 600 nm. All measurements were performed in triplicate (*n* = 3) at 25 °C.

### 2.5. Particle Size Distribution and Zeta Potential

The hydrodynamic diameter, polydispersity index (PDI), and zeta potential of the RPI-SA composite particles were determined using a Zetasizer Nano ZS (Malvern Instruments, Malvern, Worcestershire, UK) equipped with a 633 nm He-Ne laser. Prior to analysis, the samples were diluted to an RPI concentration of 1.0 mg/mL using ultrapure water adjusted to pH 5.9. Measurements were conducted immediately after dilution to minimize changes in particle aggregation and multiple scattering effects. Particle size and PDI were measured by dynamic light scattering (DLS) at a backscattering angle of 173° and 25 °C, following equilibration for 120 s. The refractive indices were set at 1.330 for the dispersant (water) and 1.450 for the particles, with an absorption index of 0.001, and the viscosity of the dispersant was assumed to be 0.8872 cP at 25 °C. The results were expressed as the intensity-weighted Z-average diameter. Zeta potential was measured by phase analysis light scattering (PALS) at a detection angle of 17° in a disposable folded capillary cell (DTS1070) and was calculated from the electrophoretic mobility using the Smoluchowski model. Each sample was analyzed in triplicate (*n* = 3), with at least 10 sub-runs per measurement, and the results are reported as mean ± standard deviation.

### 2.6. UV-Visible Spectroscopy

UV-visible absorption spectra were recorded to evaluate the conformational changes in RPI upon complexation with SA. Prior to analysis, samples were diluted with 10 mM phosphate buffer (pH 5.9) to a final RPI concentration of 1.0 mg/mL. Spectra were scanned from 200 to 400 nm at 25 °C using a spectrophotometer (UV-1100, MAPDA, China) in a 1 cm path-length quartz cuvette, with a bandwidth of 1.0 nm and a data interval of 1 nm, against the corresponding buffer as the blank. Changes in absorbance intensity and peak position near 280 nm, which arise mainly from tryptophan and tyrosine residues, were examined to assess alterations in the microenvironment of the aromatic amino acid residues. All measurements were performed in triplicate (*n* = 3).

### 2.7. Intrinsic Fluorescence Spectroscopy

Intrinsic fluorescence spectroscopy was employed to investigate the tertiary structural changes in RPI upon complexation with SA. Prior to analysis, the samples were diluted with 10 mM phosphate buffer (pH 5.9) to a final RPI concentration of 0.5 mg/mL, corresponding to an absorbance below 0.05 at the excitation wavelength, to minimize the inner filter effect. Fluorescence spectra were recorded at 25 ± 0.1 °C using a fluorescence spectrophotometer (F-4600, HITACHI Ltd., Tokyo, Japan) in a 1 cm four-sided quartz cuvette, with excitation at 290 nm and emission scanned from 300 to 420 nm. Both excitation and emission slit widths were set at 5 nm. Buffer blanks were recorded under identical conditions and subtracted from all spectra. Variations in fluorescence intensity and shifts in the maximum emission wavelength (λ_max_) were analyzed to assess alterations in the microenvironment of the tryptophan residues. All measurements were performed in triplicate (*n* = 3).

### 2.8. Surface Hydrophobicity

The surface hydrophobicity (H_0_) of the RPI/SA composite particles was determined using ANS as a fluorescent probe. Samples were diluted with 10 mM PBS to a series of RPI concentrations of 0.05%, 0.10%, 0.15%, 0.20%, and 0.25% (*w*/*v*). For each assay, a 200 μL aliquot of the diluted sample was mixed with 2 μL of ANS solution (8 mmol/L), yielding a final ANS concentration of approximately 79.2 μmol/L, and incubated in the dark at 25 °C for 3 min. Fluorescence spectra were recorded using the spectrofluorometer described in Section 2.7, with excitation at 390 nm and emission scanned from 400 to 600 nm and both slit widths set at 5 nm. The fluorescence intensity of a blank containing ANS in buffer and the background scattering of the corresponding protein solutions without ANS were subtracted from each spectrum. The surface hydrophobicity index (H_0_) was calculated as the initial slope of the linear regression of the maximum fluorescence intensity against the protein concentration (ranging from 0.05% to 0.25%, *w*/*v*), with all linear regressions exhibiting a goodness-of-fit (R^2^) greater than 0.95. All measurements were performed in triplicate (*n* = 3).

### 2.9. Foaming Properties

The foaming properties of the RPI/SA composite particle dispersions, expressed as foamability (FA) and foam stability (FS), were determined according to the method of Zhan et al. [41] with minor modifications. A 10 mL aliquot of each dispersion (1%, *w*/*v*) was placed in a 25 mm × 150 mm glass tube and homogenized at 10,000 rpm for 2 min at 25 °C using a homogenizer (T-18, IKA, Staufen, Germany). The foam volume was recorded within 30 s after homogenization (V_1_) and again after standing at 25 °C in capped tubes for 60 min (V_2_). FA and FS were calculated as follows:

$$\mathrm{FA}\left(\%\right)=\frac{V_{1}}{V_{0}}\times 100\%$$

$$\mathrm{FS}\left(\%\right)=\frac{V_{2}}{V_{1}}\times 100\%$$

where V_0_ is the initial liquid volume (10 mL), V_1_ is the foam volume immediately after homogenization, and V_2_ is the foam volume after standing for 60 min. All measurements were performed in triplicate (*n* = 3) at 25 °C, and the results are expressed as mean ± standard deviation.

### 2.10. Dynamic Interfacial Tension and Interfacial Dilatational Rheology Measurement 

Dynamic interfacial tension at the air-water interface was measured using a pendant drop tensiometer (Tracker, Teclis, Civrieux-d’Azergues, France) equipped with an oscillating drop module. A drop of RPI/SA dispersion (approximately 10 µL) was formed at the tip of a stainless-steel needle in air, and the interfacial tension (γ, mN/m) was recorded continuously for 10,800 s to ensure the protein adsorption layer reached a quasi-equilibrium state. The air phase was saturated with water vapor to minimize droplet evaporation and the temperature was maintained at 25 ± 0.5 °C using a circulating water bath connected to the measuring cell. The initial interfacial tension was recorded immediately after drop formation.

Interfacial dilatational rheology was measured using the same instrument. The oscillation frequency was 0.1 Hz and the area amplitude was 5% of the initial drop area, which was predetermined to be within the linear viscoelastic region of the interfacial film. In parallel experiments, the interfacial dilatational modulus (E, mN/m) was recorded continuously as a function of time for 10,800 s to monitor the development of the interfacial film. The interfacial dilatational modulus was calculated as:

E = dγ/dln A = Adγ/dA

where γ is the interfacial tension (mN/m) and A is the drop area (mm^2^). All interfacial rheology measurements were performed at 25 ± 0.5 °C.

## 3. Results and Discussion

### 3.1. Particle Size and Polydispersity Index of RPI/SA Composite Particles

**Figure 1:**
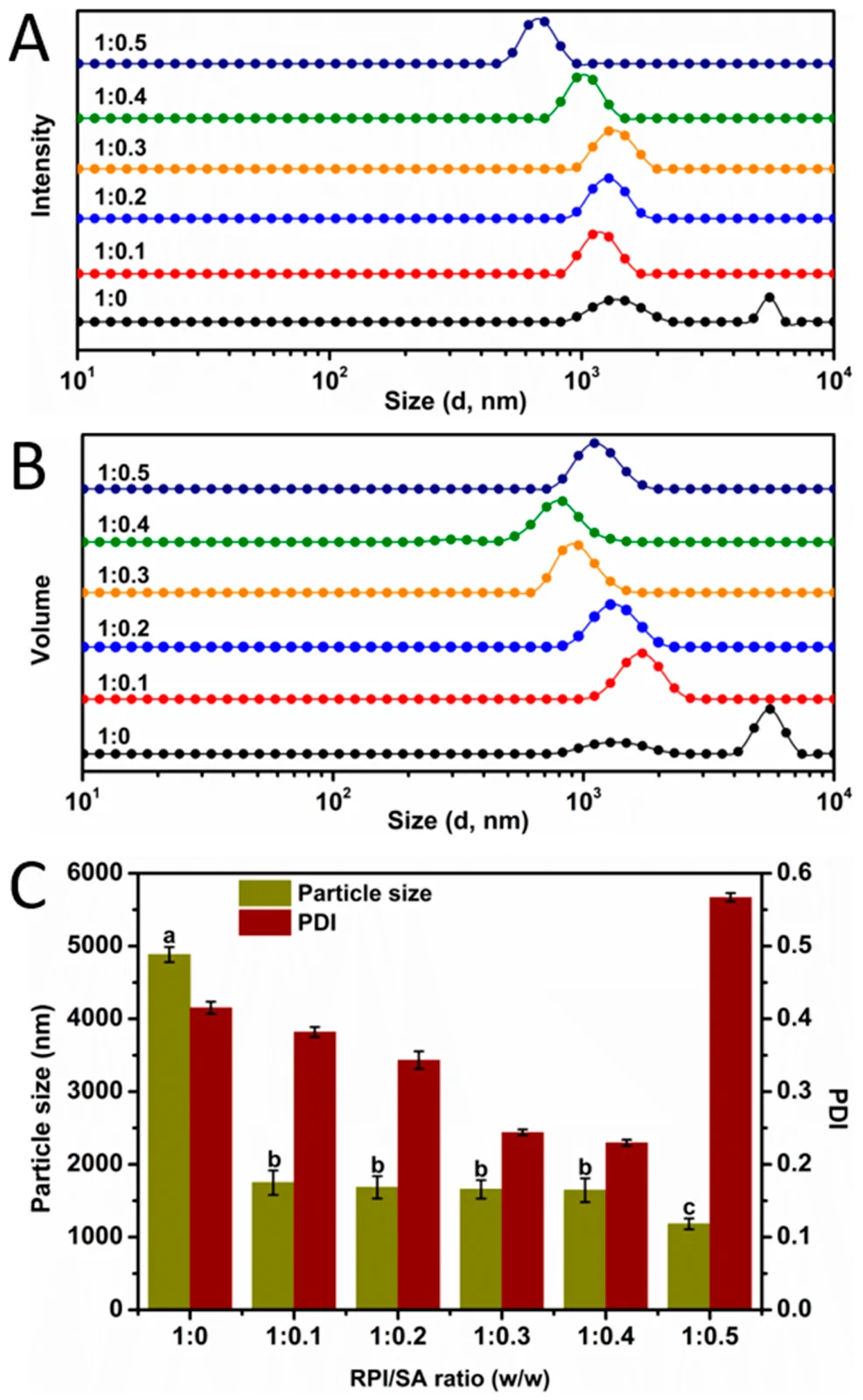
(**A**) Particle size distribution by intensity; (**B**) particle size distribution by volume; (**C**) particle size and PDI of RPI-SA complexes at different RPI/SA ratios (*w*/*w*). Data are presented as means ± standard deviation (SD). Different superscript letters (a–c) indicate significant differences (*p* < 0.05). The error bars indicate the standard deviation (SD) (*n* = 3).

Modulating the RPI/SA mass ratio significantly affected the hydrodynamic diameter and polydispersity index (PDI) of the composite colloidal systems (Figure 1). The mean hydrodynamic diameter of the complexes decreased progressively with increasing SA content. The pristine RPI system (1:0) underwent extensive thermal aggregation, yielding an apparent mean diameter of ~4900 nm after heating at 80 °C; this behavior is attributed to heat-induced protein unfolding and the subsequent intermolecular association driven by exposed hydrophobic domains [42]. The incorporation of SA effectively suppressed macroscopic aggregation, reducing the particle size to ~1100–1500 nm across all composite formulations, demonstrating that SA acts as a macromolecular chaperone promoting the formation of kinetically stable colloidal complexes.

In contrast to the monotonic reduction in particle size, the PDI—an index of distribution homogeneity—followed a non-monotonic, V-shaped profile. The system became progressively more uniform at low-to-moderate SA levels, attaining a minimal PDI of ~0.20 at an optimal RPI:SA ratio of 1:0.4 (with 1:0.3 displaying a comparably narrow distribution of ~0.22). Further increasing the SA proportion to 1:0.5, however, triggered a sharp surge in PDI to ~0.55, signifying a transition from a relatively monodisperse population to a heterogeneous, multimodal size distribution. Notably, the apparent drop in Z-average diameter (~1100 nm) at 1:0.5 alongside the elevated PDI reflects an artifact of dynamic light scattering (DLS) cumulant analysis in polydisperse systems: the coexistence of unadsorbed, fast-diffusing free SA chains in the continuous phase skews the intensity-weighted size toward smaller values, while simultaneously broadening the distribution profile.

These structural variations originate from the balance of non-covalent forces between RPI and SA during thermal processing. Upon heating, RPI partially unfolds to expose reactive binding sites. Although both biopolymers carry net negative charges at pH 5.9 (pI of RPI ≈ 4.8), SA chains electrostatically anchor onto localized positively charged patches on the RPI surface, reinforced by cooperative hydrogen bonding and hydrophobic interactions [43,44]. As a linear polyanion, the adsorbed SA increases the local surface charge density, strengthening electrostatic repulsion while simultaneously forming a thick, hydrophilic hydration shell. This combined electrostatic and steric barrier effectively suppresses the heat-induced coalescence of protein cores [32,45].

Colloidal stability reached an optimal state at RPI:SA ratios of 1:0.3 and 1:0.4, where SA chains provided adequate and uniform surface coverage. Beyond this window (1:0.5), excess polysaccharide disrupted colloidal equilibrium. This destabilization can be rationalized by three cooperative mechanisms: bridging flocculation induced by surplus SA chains tethering neighboring particles [46], chain self-entanglement that impedes uniform surface spreading [47], and depletion flocculation driven by unadsorbed SA in the continuous phase, which establishes an osmotic pressure gradient that forces localized particle clustering [48]. In summary, the RPI/SA mass ratio plays a pivotal role in balancing particle size against dispersion uniformity. The moderate hydrodynamic diameters (~1500 nm) and narrow size distributions (PDI ~0.20) achieved at ratios of 1:0.3 and 1:0.4 offer an ideal colloidal architecture for subsequent interfacial adsorption and foam stabilization.

### 3.2. Effect of RPI/SA Mass Ratio on the Zeta Potential of Composite Particles

**Figure 2:**
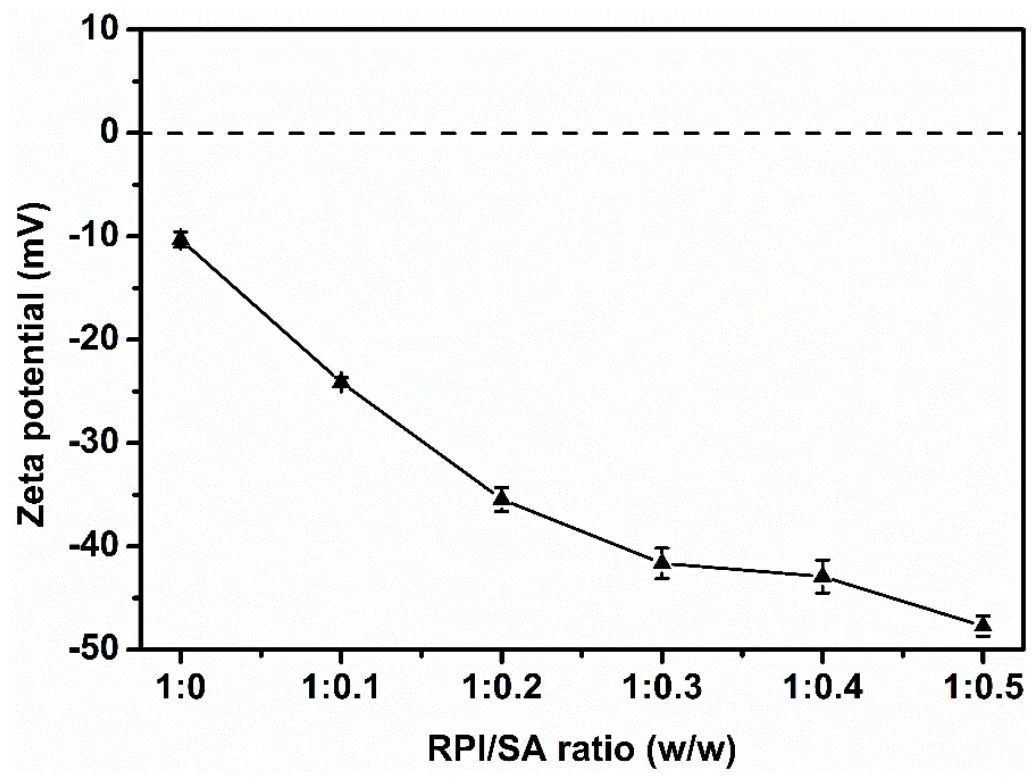
Effect of different RPI/SA mass ratios on zeta potential. The solid line represents the trend of zeta potential, and the dashed line indicates where the zeta potential is 0. The triangle markers represent the zeta potential, and the error bars indicate the standard deviation (SD).

Zeta potential characterizes interfacial charge density and electrostatic stability, reflecting the binding behavior between protein and polysaccharide. As shown in Figure 2, thermally treated RPI alone (1:0) exhibited a weak negative potential of −10.5 mV at pH 5.9. This low surface charge density provides insufficient electrostatic repulsion to counterbalance hydrophobic attraction during cooling [49], directly explaining the severe thermal aggregation (~4900 nm) observed in Figure 1.

The incorporation of SA induced a pronounced negative shift in zeta potential, reaching −48.8 mV at an RPI:SA ratio of 1:0.5. This substantial increase in net negative charge confirms the strong adsorption of polyanionic SA chains onto RPI. During heating, structural loosening of RPI exposed interior cationic residues, allowing carboxyl-rich (-COO^−^) SA chains to anchor via localized electrostatic attraction and hydrogen bonding, effectively remodeling the particle exterior into a dense, negatively charged shell [50,51].

Notably, the zeta potential exhibited a non-linear trajectory, plateauing between −42 and −43 mV at RPI:SA ratios of 1:0.3 and 1:0.4. This plateau indicates the saturation of the primary SA adsorption layer on the protein core, beyond which additional polysaccharide chains cannot directly associate with the particle surface. Although a high absolute zeta potential (∣ζ∣ > 30 mV) is traditionally associated with colloidal stability, stability in multicomponent biopolymer systems is not governed by electrostatics alone; steric hindrance, hydration forces, and non-DLVO interactions are equally critical [52]. Indeed, despite exhibiting the highest surface charge (−48.8 mV), the 1:0.5 system displayed impaired uniformity (PDI ~0.55), confirming that non-adsorbed SA chains induce non-DLVO bridging and depletion effects. Thus, the 1:0.3–1:0.4 ratios represent the optimal electrostatic window, providing robust charge repulsion without the collateral destabilization caused by excess polysaccharide.

### 3.3. Turbidity and Macroscopic Dispersion State of RPI/SA Composite Systems

**Figure 3:**
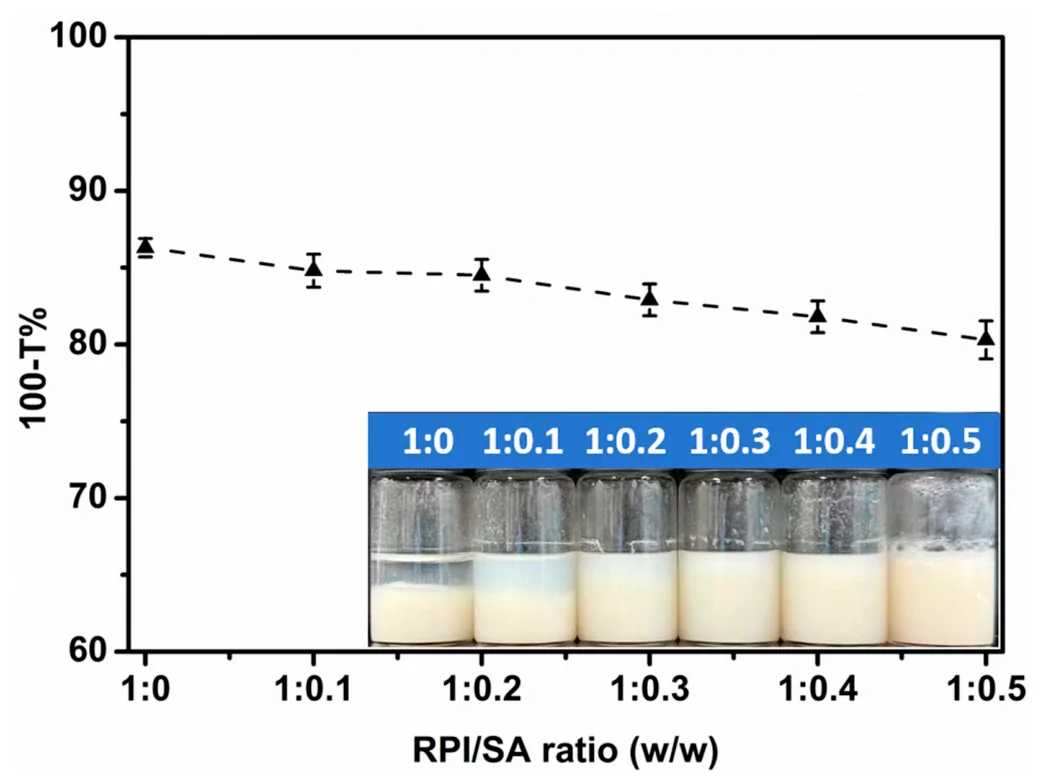
Effect of different RPI/SA mass ratios on turbidity.

Turbidity serves as a sensitive indicator of particle aggregation and macroscopic colloidal stability. As illustrated in Figure 3, the RPI:SA ratio profoundly altered system turbidity, with inset photographs displaying the macroscopic appearance after heating and cooling. Pristine RPI (1:0) exhibited acute phase separation, characterized by the rapid deposition of large, dense precipitates at the bottom of the container. This failure is ascribed to extensive thermal denaturation, where exposed hydrophobic domains drove irreversible macroscopic aggregation via hydrophobic collapse during cooling [53]. In stark contrast, SA incorporation substantially improved dispersion stability, converting the unstable suspension into visually homogeneous, milky-white colloidal dispersions with no visible sedimentation over prolonged storage, demonstrating that SA markedly enhances the thermal anti-aggregation capacity of rice protein [53].

Turbidity decreased monotonically with increasing SA content, which directly correlates with the particle size reduction shown in Figure 1: at a constant dispersed-phase concentration, smaller colloidal entities scatter light less efficiently than large flocs, leading to lower optical density. In pristine RPI, massive thermal aggregates (~4900 nm) induced severe light scattering and rapid gravitation-driven settling. The adsorption of SA chains restricted protein core growth to the sub-micron/micron scale (~1100–1500 nm) via combined electrostatic-steric repulsion [54,55], thereby attenuating scattering intensity. Importantly, this reduced turbidity does not reflect complete dissolution, but rather a structural transition from an unstable suspension of macroscopic flocs to a kinetically stable, uniform colloidal dispersion.

### 3.4. Analysis of UV-Visible Absorption Spectra

**Figure 4:**
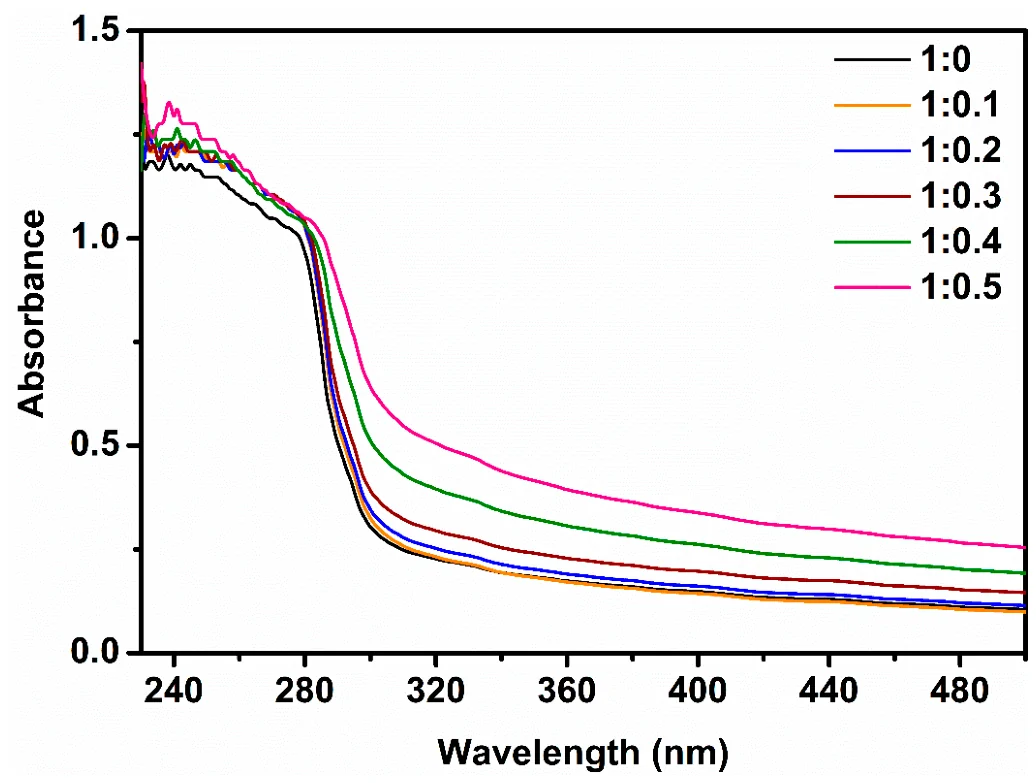
UV-visible absorption spectra of the RPI/SA composite systems.

UV–vis absorption spectroscopy was employed to assess tertiary structural variations in RPI and the microenvironmental polarity of its aromatic amino acid residues (Figure 4). The distinct absorption band between 240 and 300 nm originates from the electronic transitions of aromatic residues—tryptophan (Trp), tyrosine (Tyr), and phenylalanine (Phe) [56,57]. Because pure SA exhibits no optical absorption in this wavelength range, all spectral shifts directly mirror the conformational and dispersion state of RPI.

With increasing SA proportion, the absorbance near 280 nm exhibited a progressive, hyperchromic increase. In pristine RPI (1:0), extensive aggregation caused rapid sedimentation of large flocs out of the optical beam; simultaneously, hydrophobic collapse re-buried aromatic residues within the dense core of the remaining flocs, thereby reducing their effective absorption cross-section and leading to low absorbance [40]. In contrast, SA co-assembly preserved RPI in a highly hydrated, well-dispersed colloidal state. This prevented protein re-precipitation, keeping aromatic chromophores exposed to the solvent and sustaining a higher concentration of active absorbers in the optical path. Furthermore, the elevated baseline above 300 nm in SA-stabilized systems reflects persistent, uniform light scattering from stable colloidal particles [58], corroborating the visual appearance observed in Figure 3. The absence of any shift in the absorption peak (λ ax at ~280 nm) confirms that complexation was mediated purely by non-covalent interactions without modifying the primary chemical structure of RPI [57].

### 3.5. Intrinsic Fluorescence Spectra of RPI/SA Composite Systems

**Figure 5:**
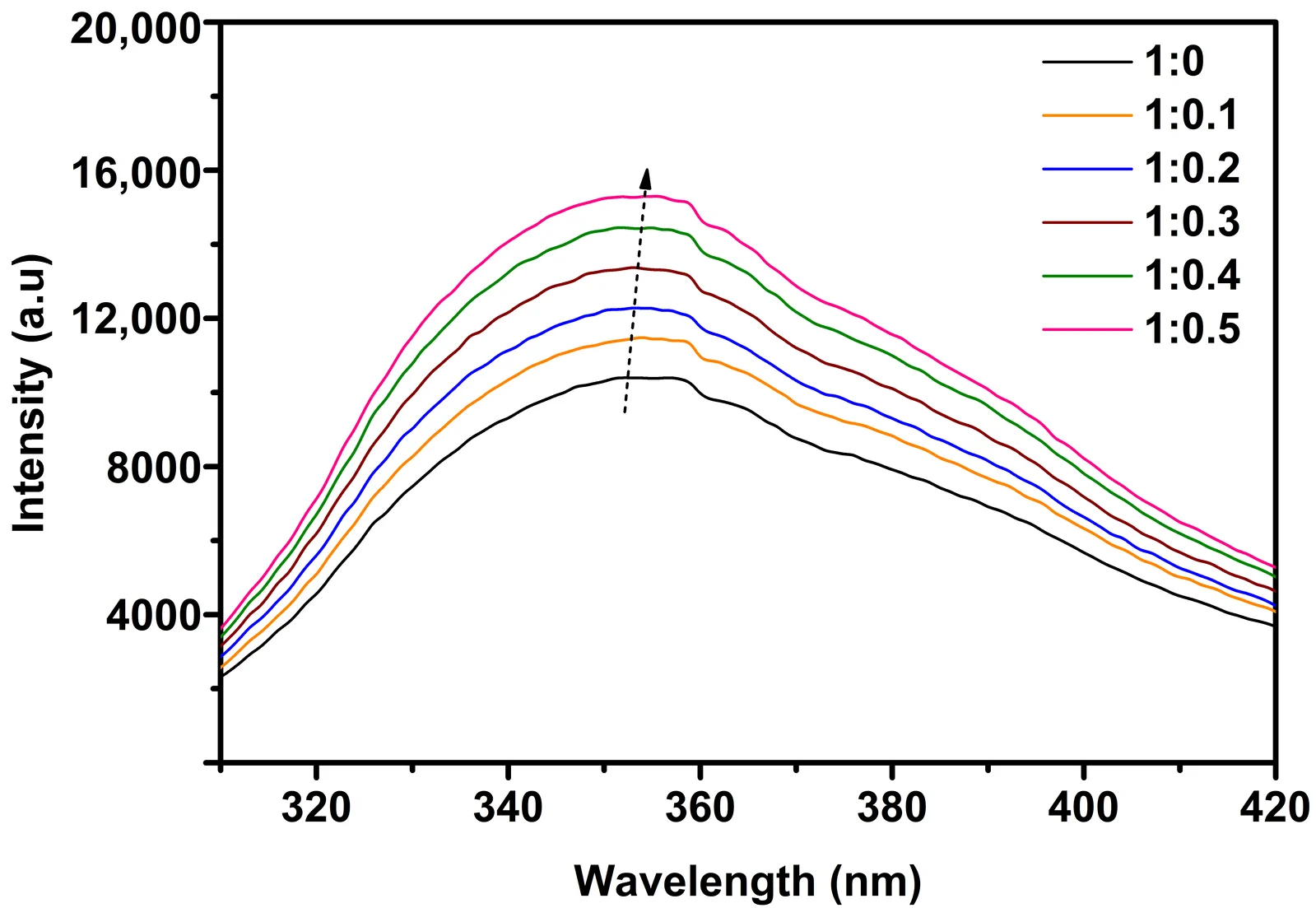
Fluorescence spectra of the RPI/SA composite systems. The dashed arrows indicate the trend of increasing fluorescence.

Intrinsic fluorescence spectroscopy was utilized to track the microenvironmental polarity around Trp residues and evaluate tertiary conformational rearrangements (Figure 5). All samples displayed a broad emission maximum centered between 350 and 360 nm. In folded globular proteins, Trp residues buried within the hydrophobic core typically emit near 330 nm, whereas direct exposure to a polar aqueous milieu shifts emission past 350 nm [56]. The emission maxima observed here (350–360 nm) confirm that thermal treatment effectively unfolded the tertiary structure of RPI, displacing Trp residues into an open, polar microenvironment—in full agreement with the UV–vis findings.

Although the emission peak positions remained unaltered across different RPI:SA ratios, the fluorescence intensity demonstrated a dramatic, SA-dependent enhancement. Pristine RPI (1:0) exhibited the lowest fluorescence intensity. This quenching stems from two cooperative effects: aggregation-caused quenching (ACQ), wherein dense molecular packing facilitates non-radiative energy dissipation through intermolecular collisions, and severe light attenuation caused by the sedimentation of macro-aggregates [59].

The introduction of SA progressively restored and heightened the fluorescence intensity. This recovery is primarily driven by the suppression of protein aggregation, which eliminates ACQ and minimizes scattering loss. Furthermore, the hydrophilic SA chains form an external hydration shell around the protein core, restricting the non-radiative vibrational and rotational dissipation of exposed fluorophores [57]. Crucially, the lack of a blue shift indicates that SA did not induce protein refolding, but rather stabilized RPI in an accessible, open conformation. These spectroscopic profiles collectively demonstrate that SA co-assembly locks RPI into a stable, non-aggregated state that favors subsequent molecular adsorption at the air–water interface.

### 3.6. Surface Hydrophobicity of RPI/SA Composite Systems

**Figure 6:**
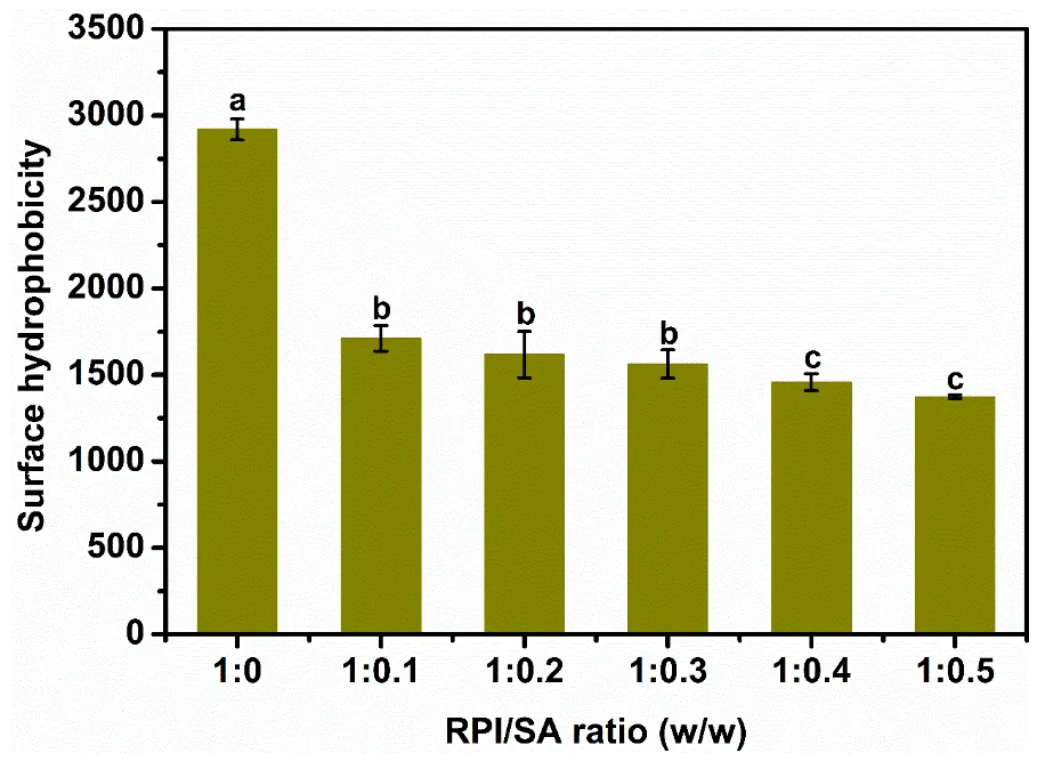
Effect of different RPI/SA mass ratios on surface hydrophobicity. Data are presented as means ± standard deviation (SD). Different superscript letters (a–c) indicate significant differences (*p* < 0.05). The error bars indicate the standard deviation (SD) (*n* = 3).

Surface hydrophobicity (H_0_) reflects the exposure of hydrophobic patches on the protein surface and dictates its thermodynamic driving force for interfacial anchoring. As shown in Figure 6, H_0_ decreased systematically with increasing SA content. Thermally treated RPI alone (1:0) exhibited the highest H_0_, corresponding to heat-induced unfolding that exposed interior hydrophobic domains. While these hydrophobic domains drove self-aggregation during cooling, a significant portion remained exposed on the aggregate surfaces, providing abundant binding sites for the ANS probe [60].

The addition of SA induced a sharp drop in H 0; even a minimal ratio of 1:0.1 reduced H_0_ to ~1700. The hydrophobicity remained relatively stable between 1:0.1 and 1:0.3, followed by a secondary decline to a minimum of ~1300 at ratios of 1:0.4 and 1:0.5. This apparent decrease does not signify an intrinsic loss of protein hydrophobicity or conformational refolding. Rather, it is primarily attributable to steric masking: the adsorbed, polyanionic SA chains construct a dense, highly hydrated outer shell that exerts substantial steric hindrance and electrostatic repulsion, thereby blocking ANS molecules from accessing hydrophobic domains on the RPI core [61]. The initial plateau between 1:0.1 and 1:0.3 aligns with the zeta potential saturation profile (Figure 2), signifying the saturation of the primary SA binding layer. At higher ratios (1:0.4–1:0.5), surplus SA chains further envelop residual hydrophobic patches via steric entanglement [56,57].

Integrating the structural, zeta potential, and hydrophobicity data, we propose a core–shell colloidal architecture for the thermally co-assembled RPI/SA particles. In this conceptual model, the particle interior comprises a partially unfolded, hydrophobic RPI-rich core, while the exterior is enveloped by a hydrophilic, negatively charged SA shell. This dual architecture balances kinetic dispersion stability in the bulk phase with interfacial anchoring capability, laying a structural foundation for high-performance foam stabilization.

### 3.7. Foaming Ability and Foam Stability of RPI/SA Composite Systems

**Figure 7:**
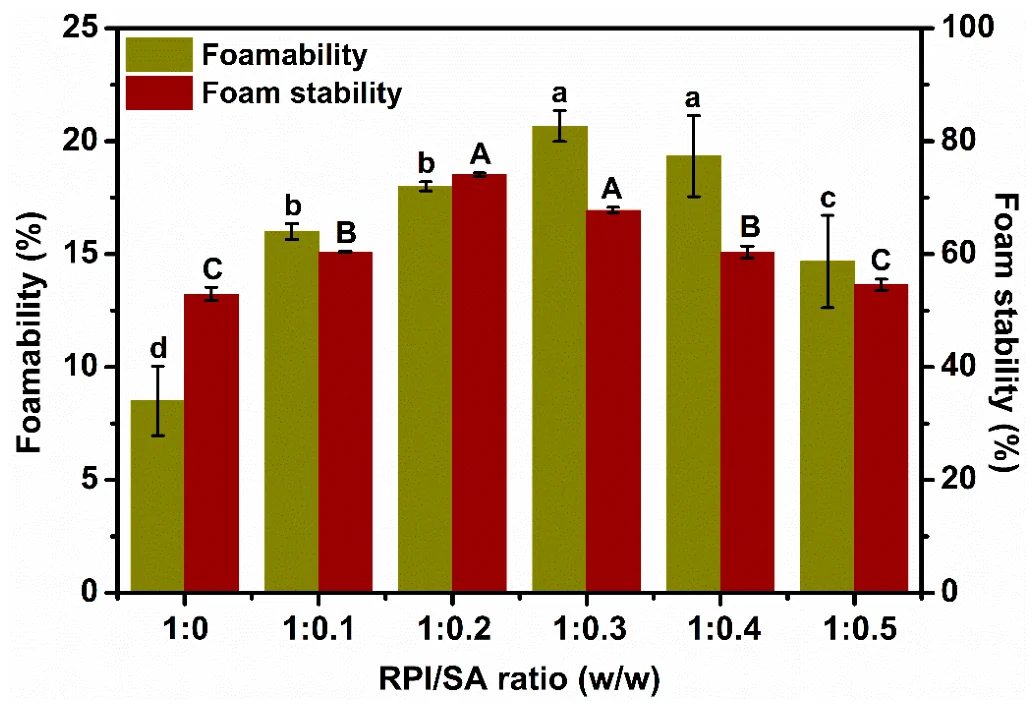
Effect of different RPI/SA mass ratios on foaming capacity and foam stability. Data are presented as means ± standard deviation (SD). Different superscript letters (A-C, a–c) indicate significant differences (*p* < 0.05). The error bars indicate the standard deviation (SD) (*n* = 3).

Foamability (FA) and foam stability (FS) were evaluated to determine the interfacial functional performance of the RPI/SA composite particles (Figure 7). Pristine RPI (1:0) exhibited exceptionally poor foaming performance, with an FA of <50% and rapid foam collapse within minutes. As evidenced by DLS and turbidity data, the massive thermal aggregates of RPI possessed low aqueous mobility and could not diffuse, adsorb, or rearrange rapidly at the newly created air-water interface during high-shear homogenization [54].

SA incorporation substantially boosted both FA and FS. Mechanistically, SA prevented thermal macro-aggregation, keeping the protein cores in a dispersed, highly active state that readily diffused to the interface, lowering interfacial tension and promoting initial bubble formation. Concurrently, the outward-facing polyanionic SA chains extended into the inter-bubble aqueous lamellae, constructing a thick hydration barrier. The resultant electrostatic repulsion and steric hindrance effectively suppressed thin-film drainage, bubble coalescence, and Ostwald ripening [62].

Remarkably, both FA and FS followed a pronounced bell-shaped curve as a function of SA content, peaking simultaneously at RPI:SA ratios of 1:0.3 and 1:0.4. This optimal functional window precisely mirrors the colloidal equilibrium identified earlier: particles at 1:0.3–1:0.4 possessed minimal PDI (~0.20), saturated negative surface charge (−43 mV), and moderate hydrodynamic dimensions (~1500 nm). Conversely, excess SA (1:0.5) led to a marked impairment in both FA and FS. This decline arises from two interrelated factors. First, excess non-adsorbed SA coils in the bulk phase elevated continuous-phase viscosity, which restricted air incorporation during blending and slowed particle migration kinetics toward the expanding bubble surfaces. Second, as evidenced by the sharp surge in PDI, excessive SA induced depletion and bridging flocculation, generating irregular, heterogeneous flocs that failed to pack cohesively at the air–water boundary. Defective, non-uniform interfacial layers are highly susceptible to localized capillary rupture and rapid coarsening [62].

### 3.8. Dynamic Interfacial Tension and Interfacial Dilatational Rheology

**Figure 8:**
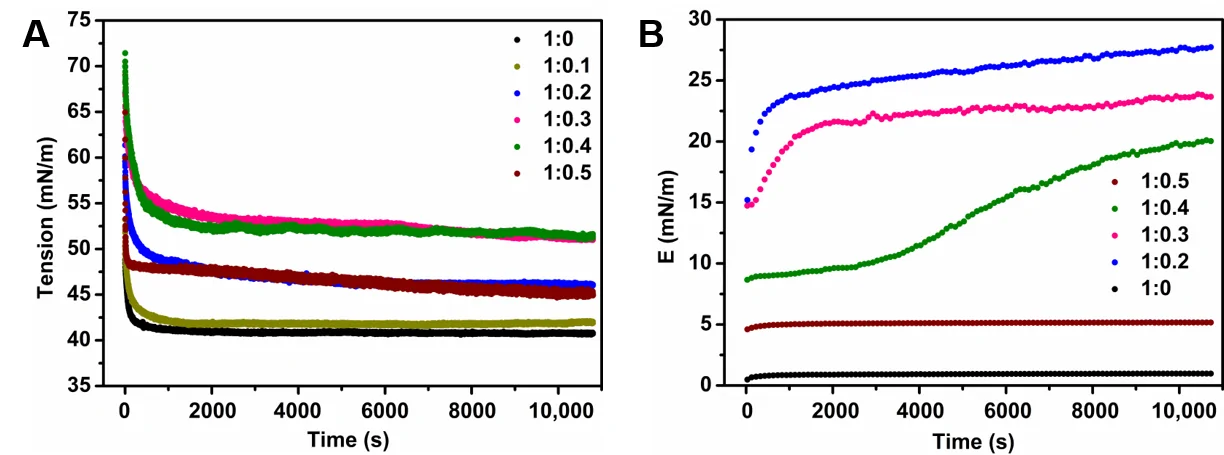
(**A**) Time evolution of the surface pressure; (**B**) surface dilatational modulus for RPI/SA composite at the air-water interface.

Dynamic interfacial tension (γ) and interfacial dilatational modulus (E) were monitored to elucidate the molecular and colloidal mechanisms governing air–water interfacial film formation (Figure 8). As shown in Figure 8A, all dispersions induced a continuous, time-dependent reduction in interfacial tension from the clean air–water boundary value (~72 mN/m) toward a quasi-equilibrium plateau, confirming the active interfacial adsorption of colloidal species. Notably, pristine RPI (1:0) and the 1:0.1 complex reached the lowest plateau tensions (~40–41 mN/m), indicating rapid interfacial migration driven by abundantly exposed hydrophobic patches. However, despite reaching low interfacial tension, pristine RPI produced an exceptionally fragile interfacial film, displaying a negligible dilatational modulus (E < 1 mN/m; Figure 8B) [63]. This contrast highlights a classic interfacial paradox: rapid surface coverage and low interfacial tension do not necessarily translate into a mechanically cohesive film, explaining why pristine RPI failed to stabilize foam [64].

SA co-assembly markedly reinforced the mechanical integrity of the interfacial layer. The 1:0.2–1:0.4 complexes developed the highest dilatational moduli (E ≈ 18–24 mN/m), denoting the formation of a highly viscoelastic, cross-linked interfacial network [32]. Interestingly, these optimal formulations did not yield the lowest equilibrium tensions; instead, they stabilized at higher plateau values (~50–52 mN/m). This behavior is characteristic of particulate stabilizers: the hydrophilic SA coating moderately slows final interfacial relaxation and reduces absolute interfacial pressure, yet the adsorbed particles form a dense, jammed interfacial layer that exhibits superior mechanical resistance to deformation. At an RPI:SA ratio of 1:0.5, the dilatational modulus dropped sharply to ~5 mN/m, indicating that steric hindrance from excess hydrophilic SA chains and the presence of heterogeneous depletion flocs severely disrupted uniform interfacial packing.

These rheological findings delineate the decisive role of interfacial viscoelasticity in foam behavior: while initial adsorption kinetics (tension reduction) govern bubble creation and foamability (FA), long-term foam stability (FS) is overwhelmingly dictated by interfacial elasticity (E). The engineered core–shell architecture at 1:0.3–1:0.4 strikes an optimal interfacial compromise. The sub-micron composite particles (~1500 nm) function as plant-based Pickering-like stabilizers: upon reaching the air–water interface, the partially exposed protein core provides robust anchoring, while the hydrated SA shells interlock laterally to construct a cohesive, two-dimensional viscoelastic particulate network. This particulate film provides substantial steric resistance against Laplace pressure-driven bubble coalescence and mechanical deformation, while concurrently immobilizing water in the Plateau borders to retard gravitational drainage. Consequently, tuning the RPI/SA ratio to 1:0.3–1:0.4 establishes a robust structural paradigm for maximizing plant-protein foaming performance.

## 4. Conclusions

RPI/SA composite colloidal particles were successfully fabricated via heat-induced co-assembly at pH 5.9. Incorporating SA effectively suppressed the irreversible thermal macro-aggregation of native RPI, transforming unstable precipitates into uniform colloidal particles (~1100–1500 nm) with a highly negative surface charge (−42 to −48.8 mV) and superior dispersion stability. Spectroscopic and surface hydrophobicity analyses confirmed that the composites adopt a core–shell architecture, in which the partially unfolded, hydrophobic RPI core is enveloped by a hydrated, polyanionic SA shell via localized electrostatic interactions and hydrogen bonding.

Foaming performance was governed by the RPI:SA mass ratio, following a distinct bell-shaped profile that peaked at 1:0.3 and 1:0.4. Crucially, foam stabilization was dictated by interfacial film viscoelasticity rather than equilibrium tension reduction alone. At optimal ratios (1:0.3–1:0.4), the composites achieved minimal polydispersity (PDI ~0.20) and the maximum interfacial dilatational modulus (~18–24 mN/m). These sub-micron particles functioned as effective Pickering-like stabilizers at the air–water interface, where rapid protein anchoring coupled with the lateral interlocking of the hydrated SA shells constructed a robust, viscoelastic 2D interfacial network that arrested bubble coalescence and liquid drainage. Conversely, excess SA (1:0.5) induced depletion flocculation and elevated bulk phase viscosity, compromising interfacial film continuity and impairing foaming functionality.

In summary, this work elucidates the composition–structure–foaming property relationships of RPI/SA composite particles and highlights interfacial dilatational rheology as the defining determinant of plant-based foam stability. These findings establish a practical formulation strategy for upgrading insoluble rice protein into high-performance, clean-label foam stabilizers for aerated food systems.

## Figures and Tables

**Table 1:** Formulation for the preparation of RPI-SA composite.

Component	1:0	1:0.1	1:0.2	1:0.3	1:0.4	1:0.5
RPI (mL)	2.50	2.50	2.50	2.50	2.50	2.50
SA (mL)	0.00	0.50	1.00	1.50	2.00	2.50
Ultrapure water (mL)	7.50	7.00	6.50	6.00	5.50	5.00

## Data Availability

The raw data supporting the conclusions of this article will be made available by the authors on request.
